# Supplementary material for: Transitioning the Molecular Tumor Board from Proof of Concept to Clinical Routine: A German Single-Center Analysis
Source: Cancers (Basel). 2021 Mar 8;13(5):1151. doi: 10.3390/cancers13051151 (PMC7962829; doi:10.3390/cancers13051151)
Supplement: Supplementary file 1 [file cancers-13-01151-s001.zip › cancers-1093023-supplementary.pdf]

# Supplementary materials

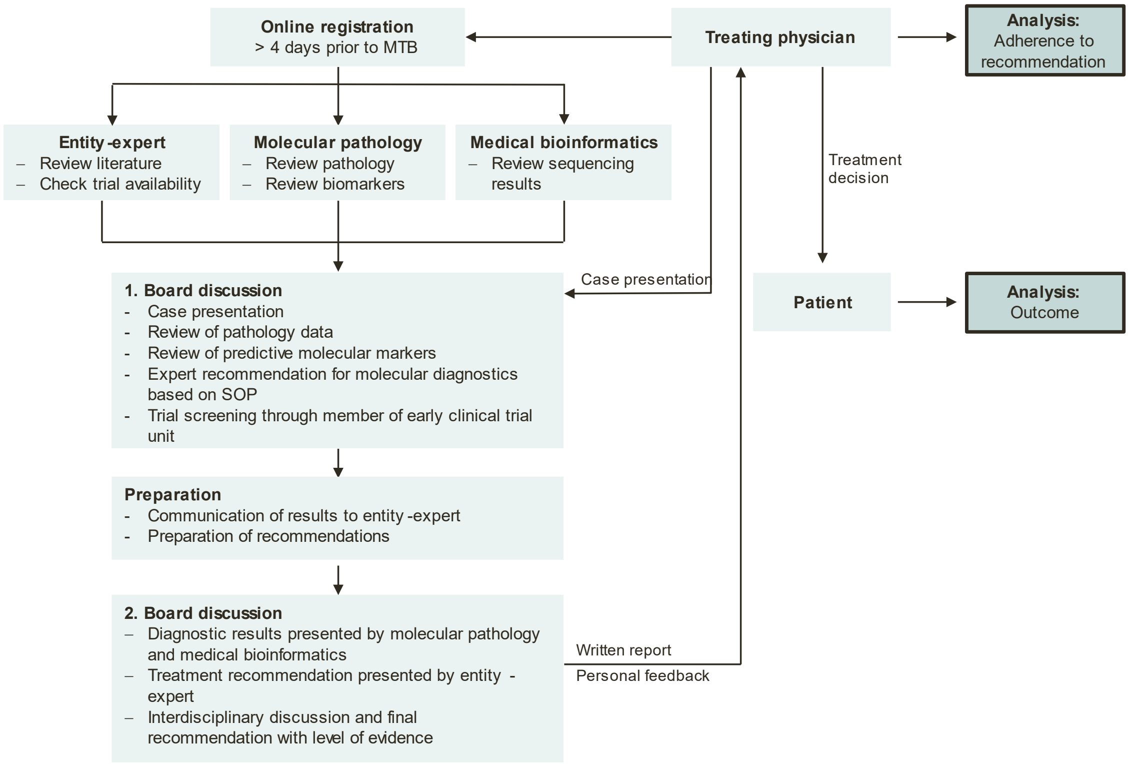


**Supplementary Figure S1.** Revised workflow of the MTB.

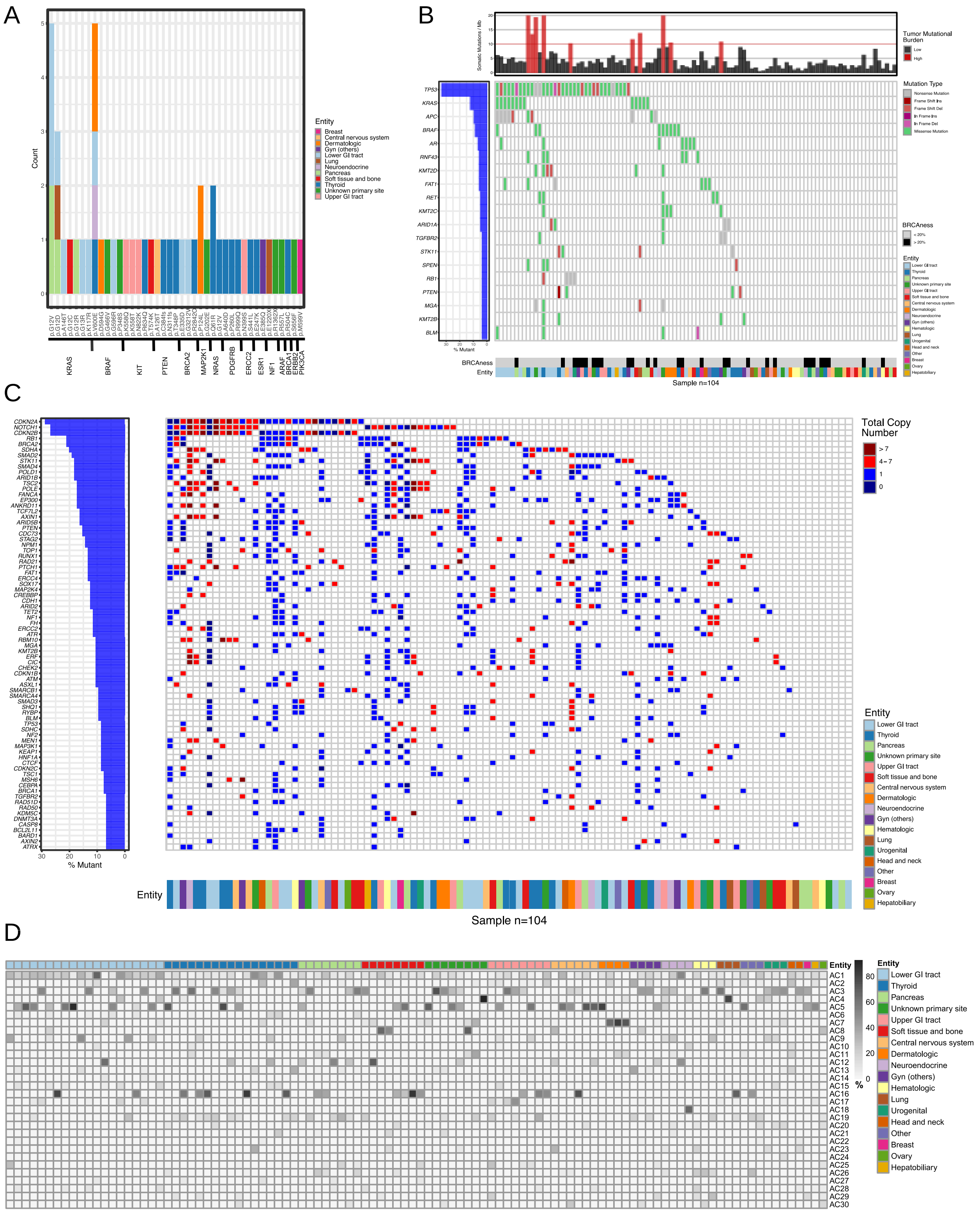


**Supplementary Figure S2.** Extended results of whole exome sequencing. (**A**) The bar diagram depicts all mutations including amino acid exchanges that were annotated as targetable by the OncoKB algorithm. The colours indicate tumor entities. (**B**) The heatmap depicts the most frequently mutated somatic cancer

genes identified by WES. The colours indicate tumor entities, type of mutation, tumor mutational burden and BRCAness-score. Only mutations with a variant allele frequency greater than 10% and a minor allele frequency less than 0.1% were considered. (**C**) The heatmap depicts copy number variations of the most frequently affected tumor suppressor genes. The colours indicate tumor entities and the total copy number per tumor suppressor gene. (**D**) Mutational signature of sequenced tumors. The colours indicate tumor entities and the intensity of grey correlates with the percentage of a given signature.

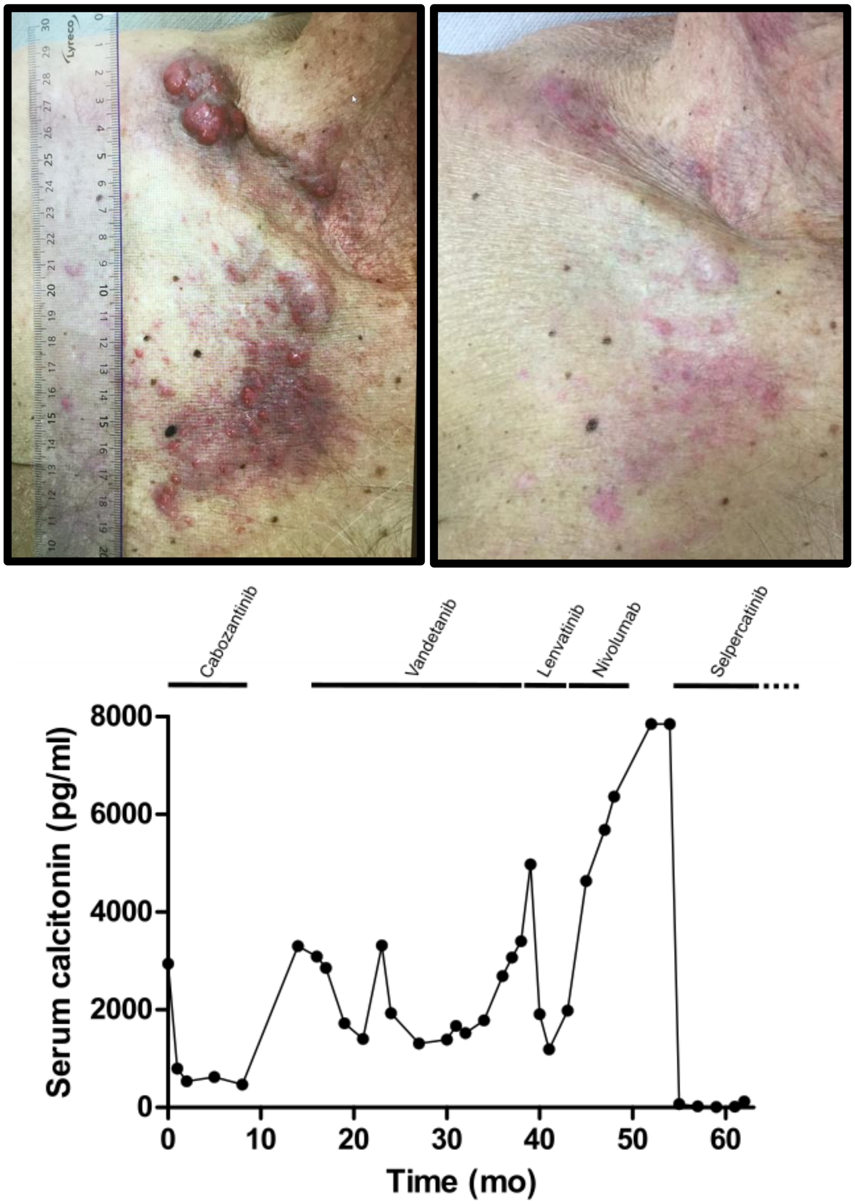


**Supplementary Figure S3.** Targeting RET in medullary thyroid cancer. (**A**) Left picture shows progressive cutaneous metastases before the treatment initiation of selpercatinib. The picture on the right shows the complete regression of the cutaneous metastases after 20 weeks on treatment. (**B**) Serum calcitonin levels under the five systemic treatments as indicated.

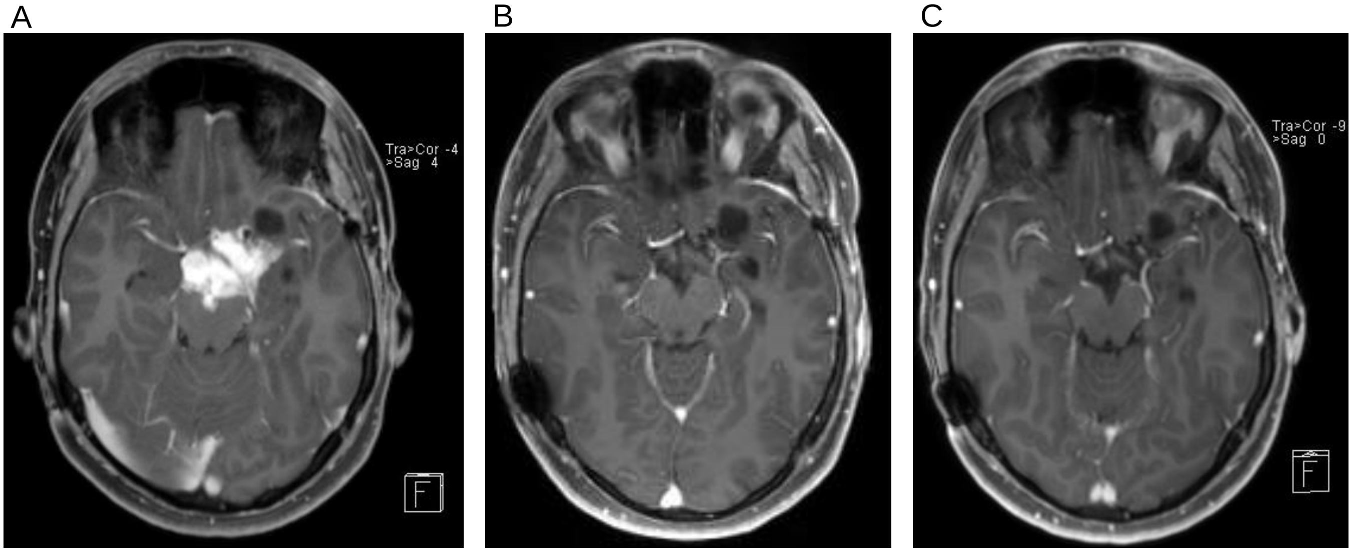


**Supplementary Figure S4.** Targeting BRAF/MEK in pleomorphic xanthoastrocytoma. MR staging in T1 sequence with contrast medium: (**A**) Left picture shows progressive tumor with contrast medium uptake four months after neurosurgical debulking. (**B**) Middle picture shows partial remission with minor uptake of contrast medium four months after treatment initiation with dabrafenib and trametinib. (**C**) Right picture shows complete remission of the contrast medium uptaking tumor after 16 months of systemic treatment.

**Case reports:** Successful molecularly guided therapy through the MTB.

**Case 1:** This is the encouraging course of a 77-year-old man fighting against metastasized medullary thyroid cancer for more than three decades. Upon initial diagnosis in 1984 he underwent surgery and radiation therapy. The cancer progressed in the same year, and he underwent multiple metastasectomies until 2010. A cerebral metastasis in 2011 was treated with radiation therapy using a linear accelerator. In the following years he experienced slow but constant tumor progression that was further treated with multiple surgical resections and multiple unsuccessful naturopathy procedures (i.e., mistletoe therapy, intratumoral injection of microbial preparations and hyperthermia treatment). Due to clinical progression and a rise of serum calcitonin to 2942 pg/ml he presented to our oncology department in June 2015. Under his first systemic treatment with cabozantinib (80 mg/d) calcitonin decreased rapidly to 462 pg/ml within four weeks (Supplementary Figure 1b) but dosage had to be reduced (40 mg/d) due to grade 3 toxicity (mucositis, hypocalcaemia, fatigue) and treatment was eventually discontinued. Calcitonin increased to 3305 pg/ml and CT staging showed progressive cervical and thoracic metastases. We started systemic treatment with vandetanib in May 2017 that led to a partial response (calcitonin 1526 pg/ml) and was better tolerated. Eventually the patient again suffered from significant tumor progression (calcitonin 4974 pg/ml) in August 2018. We switched the treatment to lenvatinib resulting in a fast response of calcitonin (1189 pg/ml in October 2018). Unfortunately, the patient was again suffering from grade 3 toxicity (mucositis, fatigue, and diarrhoea). He was referred to the MTB due to lack of further systemic therapy options. The MTB recommended a re-biopsy that was performed in September 2018 from a lymph node metastasis from the axilla. Targeted NGS confirmed a gain-of-function, oncogenic RET p.Met918Thr mutation, that had previously been detected in a tumor sample from 2013. No other potential driver mutation was identified. The IO-panel showed negative PD-L1 expression on cancer cells and weak expression of PD1 on a few intratumoral lymphocytes. Trial screening revealed an available ICB-study on-site, testing nivolumab in rare cancer (NCT02832167), and a phase I/II study of a new RET-inhibitor (LOXO-292, selpercatinib) for patients with advanced solid tumors (NCT03157128) available at the University clinic of Cologne (Germany). The patient decided first to stay on-site and was enrolled in the ICB-study. He received the first dose of nivolumab in October 2019. The first CT-staging after three courses of nivolumab in December 2018 showed a stable disease. The second CT-staging after a total of 7 cycles revealed progressive disease of the cervical and thoracic metastases in line with increasing serum calcitonin to 5500 pg/ml. However, the patient still experienced significant clinical benefit reflected by improved performance status (ECOG 1 vs. 3) and reduced neck pain. We therefore continued the nivolumab treatment within the study and beyond progression for additional seven cycles until cutaneous metastases progressed rapidly and calcitonin increased to 6329 pg/ml in July 2019 (Supplementary Figure 1a and b). Meanwhile we initiated a trial screening for the above mentioned RET-inhibitor study in Cologne. The trial screening failed due to the long half-life period of nivolumab. However, we applied to an extended access program of selpercatinib and were able to receive the drug directly from the company. We initiated the treatment (selpercatinib 160 mg twice daily) in October 2019. Within three weeks calcitonin level decreased to 70 pg/ml and cutaneous metastases responded promptly. First CT-staging in February 2020 also revealed partial remission. Side effects were hypertension (Grade 2, controlled with amlodipine) and fatigue (Grade 2). The later led to dose reduction to 80 mg twice daily in June 2020. Till the day of the preparation of the manuscript treatment and remission are ongoing (January 2021).

**Case 2:** This is the course of a 25-year-old male who got the initial diagnosis of pilocytic astrocytoma grade 1 in 2011. After interstitial stereotactic radiosurgery he experienced a relapse that was treated with proton beam therapy in 2013. This stabilized the clinical situation until July 2018. Due to a large relapse in the frontotemporal lobe, he underwent neurosurgical debulking of the tumor. Histology revealed the diagnosis of a pleomorphic xanthoastrocytoma grade 2. Unfortunately, MR staging four months after surgery showed

progressive disease of the remaining tumor with marked contrast medium uptake in the pre-mesencephalic area (Supplementary Figure 2A). The patient was referred to the MTB in December 2018 and tNGS detected a BRAF-V600E mutation. Based on published case reports [53,54] we recommended a BRAF/MEK double blockade with dabrafenib and trametinib. Treatment was initiated in January 2019 and first MR staging in May 2019 showed very good partial remission of the tumor with only minor uptake of contrast medium (Supplementary Figure 2A). The latest staging in November 2020 showed a complete remission without uptake of contrast medium and only minor remaining cystic lesions compatible with post therapy defects (Supplementary Figure 2C). No side effects of the systemic treatment were observed. Till the day of the preparation of the manuscript treatment and remission are ongoing (January 2021).

**Supplementary Procedures:** Diagnostic standard operation procedures

**Therapy related biomarkers for all patients:**

**Immunooncology Panel (IO-Panel)**

PDL1, PD1, TILs (CD3, CD4, CD8)

**DNA Mismatch Repair Testing (MMR)**

MLH1, PMS2, MSH2, MSH6

**Microsatellite Instability Testing (MSI)**

Adequate multiplex PCR

**NTRK Gene Fusion Testing**

NTRK-1, -2, -3 via IHC or Fusion-Panel

**Entity specific biomarkers:**

**Breast cancer**

POLE if MSI negative, PTEN, CDK4, Cyclin D1, pS6, tNGS

Triple negative: additionally Androgen receptor

**Cancer of unknown primary**

ALK, ROS, POLE if MSI negative, Her2/neu, WES/Transcriptome

**Cholangiocarcinoma**

POLE if MSI negative, Her2/neu, tNGS

**Glioblastoma**

MSI, POLE if MSI negative, tNGS

**Head and neck cancer**

Depending on localization and histology: EGFR, Her2/neu, androgen receptor, IDH2, HPV16/18, tNGS

**Intestinal cancer**

POLE if MSI negative, Her2/neu, MET-Amplification. tNGS. Dependent on the results WES

**Lung cancer**

NSCLC: tNGS, depending on results WES/RNA-Seq

SCLC: POLE if MSI negative, PTEN, tNGS

**Melanom**a

tNGS

**Meningioma**
POLE if MSI negative, Somatostatin receptor, VEGFR1/2, ER, Gli1, tNGS

**Neuroendocrine carcinoma**
POLE if MSI negative, tNGS

**Ovarian cancer**
POLE if MSI negative, VEGFR1/2, EGFR, Her2/neu, pERK, CDK4, tNGS

**Pancreatic cancer**
POLE if MSI negative, Her2/neu, tNGS

**Pediatric tumors**
Desmoplastic small-round-cell tumor: POLE if MSI negative, VEGFR2, tNGS
Anaplastic ependymoma: pS6, MSI, POLE if MSI negative, 48 gene panel
Hepatoblastoma: EGFR, pS6, WES/RNA-Seq

**Prostate cancer**
POLE if MSI negative, tNGS, depending on results WES

**Renal cell carcinoma**
ccRCC, chRCC: PTEN, tNGS
cdRCC: Fumarate Hydratase (FH), tNGS
pRCC: MET, tNGS

**Soft tissue sarcoma**
POLE if MSI negative, VEGF, tNGS
PECOM: additionally pS6 IHC and TSC1/2 Seq.
Rhabdomyosarcoma: additionally ALK

**Thyroid cancer**
anaplastic: POLE if MSI negative, WES
papillary: POLE if MSI negative, tNGS
follicular: POLE if MSI negative, PTEN, VEGFR1/2, tNGS
mixed papillary-follicular: POLE if MSI negative, PTEN, VEGFR1/2, tNGS
medullary: RET, if RET-TKI-resistance: POLE if MSI negative. WES

**Transition cell carcinoma**
POLE if MSI negative, Her2/neu, tNGS. Dependent on the results WE

**Supplementary Table S1.** Levels of evidence

| | | |
|---|---|---|
| **Same tumor entity** | **m1A** | Predictive value or clinical effectiveness of the biomarker was demonstrated in a biomarker stratified cohort of an adequately powered prospective study or a meta-analysis. |
| | **m1B** | Predictive value or clinical effectiveness of the biomarker was demonstrated in a retrospective cohort or a case-control study. |
| | **m1C** | One or more case reports |
| **Different tumor entity** | **m2A** | Predictive value or clinical effectiveness of the biomarker was demonstrated in a biomarker stratified cohort of an adequately powered prospective study or a meta-analysis. |
| | **m2B** | Predictive value or clinical effectiveness of the biomarker was demonstrated in a retrospective cohort or a case-control study. |
| | **m2C** | One or more case reports |
| ***In vitro* or *in vivo*** | **m3** | Preclinical data (*in vitro*/*in vivo* models, functional genomics) show associations of the biomarker with the effectiveness of the recommended treatment |
| **Biologic rationale** | **m4** | Biological rationale suggests a link of the biomarker to the effectiveness of the recommended treatment. No reported preclinical data on the response to the drug. |

**Supplementary Table S2.** Details of tNGS gene panels

**Gene list of 8-gene panel**

| Gen | OMIM | Exon/s |
|---|---|---|
| BRAF | 164757 | 15 |
| EGFR | 131550 | 18,19,20,21 |
| HER2 | 164870 | 20 |
| KRAS | 190070 | 2, 3, 4 |
| KIT | 164920 | 9, 11, 13, 17 |
| NRAS | 164790 | 2, 3 |
| PDGFRa | 173490 | 18 |
| PI3KCA | 171834 | 9, 20 |

**Gene list of 15-gene panel**

| | | |
|---|---|---|
| AKT1 | GNA11 | NRAS |
| BRAF | GNAQ | PDGFRA |
| EGFR | KIT | PIK3CA |
| ERBB2 | KRAS | RET |
| FOXL2 | MET | TP53 |

**Supplementary Table S3.** Diagnostic and treatment recommendations

| | No. | (%) |
|---|---|---|
| **Total diagnostic recommendations (per patient average)** | **762 (1.6)** | |
| **Diagnostic recommendations, not implemented** | **147** | **(19.3)** |
| - Technical reasons | 66 | (44.9) |
| - Medical reasons | 26 | (17.7) |
| - Patient death | 27 | (18.4) |
| - Loss to follow-up | 14 | (9.5) |
| - Patients will | 12 | (8.2) |
| - Other | 2 | (1.4) |
| **Implemented routine molecular diagnostics** | **3550** | |
| - Immunohistochemistry | 2599 | (73.2) |
| - Targeted next generation sequencing | 412 | (11.6) |
| - In situ hybridization | 227 | (6.4) |
| - Microsatellite instability testing | 171 | (4.8) |
| - Sanger sequencing | 141 | (4.0) |
| **Total treatment recommendations** | **367** | |
| - Single agent targeted therapy | 159 | (43.3) |
| - Immune checkpoint blockade | 102 | (27.8) |
| - Combination therapy | 92 | (25.1) |
| - Chemotherapy | 8 | (2.2) |
| - Hormone therapy | 3 | (0.8) |
| - Nuclear medicine therapy | 2 | (0.5) |
| - Other | 1 | (0.3) |
| **Type of treatment recommendation** | | |
| - Off-label | 248 | (67.6) |
| - In-label | 52 | (14.2) |
| - Study | 67 | (18.3) |
| **Level of evidence** | | |
| - m1A | 62 | (16.9) |
| - m1B | 28 | (7.6) |
| - m1C | 76 | (20.7) |
| - m2A | 34 | (9.3) |
| - m2B | 18 | (4.9) |
| - m2C | 40 | (10.9) |
| - m3 | 101 | (27.5) |
| - m4 | 4 | (1.1) |
| - n.a. | 4 | (1.1) |

**Supplementary Table S4.** Details of treatment recommendations

| **Type of implemented treatment recommendation (off-label)** | **82 (62)** | **(%)** |
|---|---|---|
| - Single agent targeted therapy (off-label) | 28 (20) | (34.1) |
| - Immune checkpoint blockade + INF (off-label) | 27 (22) | (32.9) |
| - Combination therapy (off-label) | 20 (16) | (24.4) |
| - Chemotherapy (off-label) | 4 (0) | (4.9) |
| - Hormone therapy (off-label) | 3 (3) | (3.7) |
| **Implemented treatment recommendation based on** | **82** | |
| - In-label treatment | 21 | (25.6) |
| - Routine pathology – tNGS | 18 | (22.0) |
| - Routine pathology – IHC | 17 | (20.7) |
| - Extended genetic analysis | 12 | (14.6) |
| - Entity | 10 | (12.2) |
| - Study | 4 | (4.9) |
| **Patients with treatment recommendations, not implemented** | **188** | |
| - Future recommendation | 44 | (23.4) |
| - Medical reasons | 43 | (22.9) |
| - Patient death | 35 | (18.6) |
| - Loss to follow-up | 32 | (17.0) |
| - Patients will | 16 | (8.5) |
| - Declined by company or health care provider | 8 | (4.3) |
| - Study screening failure | 7 | (3.7) |
| - Other | 3 | (1.6) |
| Alternative recommendations | 103 | |

**Supplementary Table S5.** Immunooncology panel and ICB-treatment response

| Cancer type | TPS | Response |
|---|---|---|
| **Thyroid** *(anaplastic)* | 80 | CR |
| *(anaplastic)* | 5 | CR |
| *(anaplastic)* | 5 | PR |
| *(anaplastic)* | 80 | NA |
| *(anaplastic)* | 80 | PR |
| *(anaplastic)* | 30 | PR |
| *(anaplastic)* | 60 | SD |
| *(papillary)* | 80 | PR |
| *(medullary)* | 0 | SD |
| **Lower GI** *(CRC)* | 5 | PR |
| *(CRC)* | 5 | PR |
| *(CRC)* | 2 | NA |
| *(CRC)* | 0 | PD |
| *(anal)* | 0 | PD |
| *(appendix)* | NE | PD |
| **Adrenocortical** | 2 | NA |
| | 0 | PD |
| **Soft tissue** *(Sarcoma)* | 5 | PD |
| *(Sarcoma)* | 0 | SD |
| *(Desmoplastic small-round-* | 70 | PD |
| **Mesothelioma** | 0 | SD |
| **Transitional cell** | 0 | NA |
| **Gliosarcoma** | 20 | PD |
| **Laryngeal** | 1 | PD |
| **Cholangiocarcinoma** | 10 | PD |
| **Cancer of unknown** | 5 | SD |
| **Small cell lung** | 0 | PR |
| **Triple negative breast** | NA | PD |

TPS: Tumor Proportion Score. CR: complete response. PR: partial response SD: stable disease. NA: not applicable.
